# Sequence and structural features of binding site residues in protein-protein complexes: comparison with protein-nucleic acid complexes

**DOI:** 10.1186/1477-5956-9-S1-S13

**Published:** 2011-10-14

**Authors:** M Michael Gromiha, N Saranya, S Selvaraj, B Jayaram, Kazuhiko Fukui

**Affiliations:** 1 Department of Biotechnology, Indian Institute of Technology Madras, Chennai 600 036, Tamilnadu, India; 2 Computational Biology Research Center (CBRC), National Institute of Advanced Industrial Science and Technology (AIST), 2-4-7 Aomi, Koto-ku, Tokyo 135-0064, Japan; 3 Department of Bioinformatics, Bharathidasan University, Tiruchirapalli 620024, Tamilnadu, India; 4 Department of Chemistry and Supercomputing Facility for Bioinformatics and Computational Biology, Indian Institute of Technology Delhi, New Delhi 110016, India

## Abstract

**Background:**

Protein-protein interactions are important for several cellular processes. Understanding the mechanism of protein-protein recognition and predicting the binding sites in protein-protein complexes are long standing goals in molecular and computational biology.

**Methods:**

We have developed an energy based approach for identifying the binding site residues in protein–protein complexes. The binding site residues have been analyzed with sequence and structure based parameters such as binding propensity, neighboring residues in the vicinity of binding sites, conservation score and conformational switching.

**Results:**

We observed that the binding propensities of amino acid residues are specific for protein-protein complexes. Further, typical dipeptides and tripeptides showed high preference for binding, which is unique to protein-protein complexes. Most of the binding site residues are highly conserved among homologous sequences. Our analysis showed that 7% of residues changed their conformations upon protein-protein complex formation and it is 9.2% and 6.6% in the binding and non-binding sites, respectively. Specifically, the residues Glu, Lys, Leu and Ser changed their conformation from coil to helix/strand and from helix to coil/strand. Leu, Ser, Thr and Val prefer to change their conformation from strand to coil/helix.

**Conclusions:**

The results obtained in this study will be helpful for understanding and predicting the binding sites in protein-protein complexes.

## Background

Protein-protein interactions are important for most of the cellular processes in life. Hence, understanding the mechanism of protein-protein recognition at molecular level is of practical interest and has direct applications to functional genomics. Unravelling the mechanisms of protein-protein recognition is a fundamental problem, which would aid in function prediction and drug design.

The availability of structures of numerous protein-protein complexes in Protein Data Bank (PDB) enables researchers to analyze the binding sites in terms of amino acid composition, preference of residues, secondary structures, solvent accessibility, electrostatic patches, hydrophobic contacts, hydrogen bonding networks and so on [1-3]. The mapping of protein-protein interactions on protein sequences suggests that hotspots can be predicted from amino acid sequences [4]. The concepts of protein-protein interactions in terms of experimental techniques, databases, organization, cooperativity and prediction of protein-protein, protein-ligand and domain interactions have been reviewed in detail earlier [5-7].

Several methods have been proposed for identifying the binding sites in protein-protein complexes based on distance between two residues [8-11]. In our earlier work, we have developed an energy based approach for defining the binding sites in protein-protein complexes [12]. In this work, we have analyzed the binding site residues based on sequence and structures of protein-protein complexes. The results showed that the binding site residues have specific preferences at their vicinities and these residues are unique in protein-protein complexes. These binding site residues are more conserved than non-binding residues. In addition, several binding and non-binding residues prefer to change their conformation from helix to coil, strand to coil and coil to helix/strand. Specifically the residues Glu, Lys and Ser play important roles to conformational switching.

## Methods

### Dataset

We have developed non-redundant datasets of 153 protein-protein hetero dimer complexes from Protein Data Bank that have the sequence identity of less than 25% and solved with better than 3Å resolution [12]. In addition, we have used a benchmark dataset of 124 protein-protein complexes to validate our results [13]. For comparison, we have utilized a set of 81 protein-RNA complexes [14] and 212 protein-DNA complexes [15].

### Identification of binding site residues

We have calculated the interaction energy between all pairs of atoms in protein-protein complexes using AMBER force field [16]. The interaction energies of all the atoms in a residue have been summed up to assign the interaction energy of a residue. The amino acid residues with interaction energy less than -1 kcal/mol are treated as binding site residues [17].

### Binding propensity

The binding propensity (P_bind_) for the 20 amino acid residues in protein-protein complexes is defined as the ratio between the frequency of occurrence of amino acid residues in the binding sites (f_b_) and in the protein as a whole (f_t_). It is calculated using the equation:

P_bind_(i) = f_b_(i)/f_t_(i) (1)

where, i represents each of the 20 amino acid residues.

### Influence of neighboring residues

We have analyzed the influence of neighboring residues of binding sites on various aspects: (i) *B, where * is any residue and B is a binding site residue, (ii) B* and (iii) *B*, which is a tripeptide with the binding site residue at the middle.

### Conservation score

We have used the program AL2CO for computing the conservation score for all the residues in receptors and ligands in protein-protein complexes [17]. The target sequence has been compared with non-redundant sequences in SWISS-PROT [18] and multiple sequence alignment has been performed with ClustalW program [19]. The aligned sequences have been utilized to compute the conservation score for all the amino acid residues.

### Conformational switching upon complex formation

We have computed the secondary structures of all the residues in free proteins and complexes in a set of 124 protein-protein complexes [13] using DSSP [20]. The secondary structures have been assigned as helix, strand and coil. We have analyzed the conformational changes of residues based on their locations in secondary structures, preferred amino acid residues and binding site residues.

## Results and Discussion

### Occurrence of amino acid residues at various ranges of interaction energies

We have identified the binding sites in protein-protein complexes based on interaction energy as explained in Methods section. We observed that 13.9% of the residues have the interaction energy of < -1 kcal/mol and are identified as binding sites in a set of 306 proteins. We have compared the results with those obtained with distance based criteria for defining binding site residues and the data are presented in Table 1. We noticed that only 28% residues are common to each other and the percentage of binding site residues is a balance between those identified with different cutoff distances, indicating the importance of considering the energy between different atoms to define the binding residues. In addition, 5.7% of the residues have strong repulsive energies and all these residues have been identified as binding residues in distance based criteria, which are not probable binding residues in protein-protein complexes.

**Table 1:** Number and percentage of binding site residues using different methods

Criterion	Cutoff	N_bind_	%_bind_	Reference
Energy	<1 kcal/mol	5255	10.8	present work
C_α_ distance	6Å	1972	4.0	Keskin et al. [24]
C_β_ distance	6Å	3449	7.1	Glaser et al. [25]
Heavy atoms	5Å	6644	13.6	Li et al. [26]

### Conservation score for binding site residues in protein-protein complexes

We have computed the conservation score for all the residues and noticed that the binding residues are highly conserved in protein-protein complexes. This observation is consistent with earlier studies reported in the literature [21]. We have estimated the performance of conservation score for identifying the binding sites. We found that the conservation score alone could predict the binding sites at an average accuracy of 58% with a trade-off between sensitivity and specificity of 59% and 57%, respectively.

### Binding propensity of residues in protein complexes

**Figure 1:**
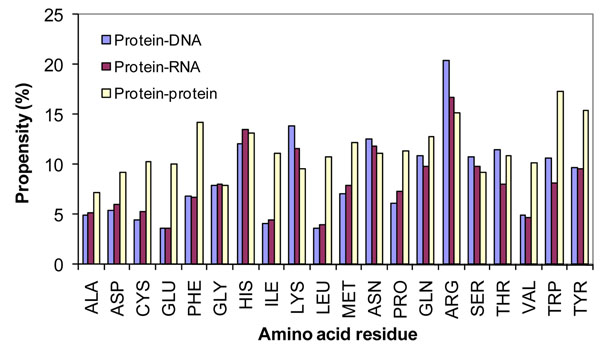
Binding propensity of amino acid residues in protein-protein, protein-RNA and protein-DNA complexes

We have computed the binding propensity in protein-protein complexes and the results are presented in Figure 1. For comparison we have also included the data obtained for protein-RNA and protein-DNA complexes.

We observed that the aromatic as well as positively charged residues highly contribute to interact between the partners in protein-protein complexes, indicating the importance of cation-π, aromatic and electrostatic interactions. The comparison between protein-protein and protein-RNA complexes showed that the residues, Asp, Cys, Glu, Phe, Ile, Leu, Met, Val, Trp and Tyr have remarkably high contribution in protein-protein complexes. These residues prefer to form electrostatic, hydrophobic and aromatic interactions in protein-protein complexes. On the other hand, the residues Arg, His, Lys, Asn, Tyr, Gln and Ser highly contribute for the interaction between protein and RNA. Interestingly, these residues belong to positively charged, polar and aromatic groups, which form electrostatic, hydrogen bonds and aromatic interactions with RNA. In protein-DNA complexes, positive charged residues are more dominant than protein-protein and protein-RNA complexes. Further, polar residues prefer to interact with DNA in the form of hydrogen bonds.

### Preference of tripeptides in the vicinity of binding sites

We have set up the following criteria to identify the probable tripeptides for binding: (i) there should be at least three samples and the probability of being in binding sites should be more than 50%. These conditions yielded a set of 208 unique tripeptides among 8000 possibilities. The usage of these tripeptides could predict the binding sites with an accuracy of 72.3% and the coverage of 6% of binding sites. The results for selected tripeptides are presented in Table 2.

We noticed that the central residue of all the tripeptides CWA, HHE, MNF and WFE are identified as binding site residues. The tripeptides LFP and MRR showed a preference of more than 75%. Although the preference is 66.7% for ITG it has the occurrence of 12 hits and eight of them are binding site residues. The preference is significantly higher than the random choice of 1.3% (10202 binding sites and the total of 8000 tripeptides).

**Table 2:** Preferred tripeptides at the binding sites of protein-protein, protein-RNA and protein-DNA complexes

Tripeptide			N_b_	N_t_	%bind
**Protein-protein**					
CYS	TRP	ALA	3	3	100.00
HIS	HIS	GLU	3	3	100.00
MET	ASN	PHE	3	3	100.00
TRP	PHE	GLU	3	3	100.00
ILE	TYR	GLY	8	12	66.67
LEU	PHE	PRO	5	6	83.33
MET	ARG	ARG	4	5	80.00
**Protein-RNA**					
GLY	TYR	GLY	3	3	100.00
PRO	GLY	ARG	3	3	100.00
ASP	LYS	TYR	6	8	75.00
GLY	SER	THR	3	4	75.00
ILE	TYR	LYS	8	12	66.67
LYS	SER	ARG	3	4	75.00
PRO	HIS	HIS	3	4	75.00
SER	ARG	LYS	5	7	71.43
VAL	GLY	SER	6	8	75.00
TYR	LYS	HIS	5	6	83.33
**Protein-DNA**					
HIS	ARG	SER	3	3	100.00
SER	GLN	THR	4	4	100.00
SER	TYR	GLN	3	3	100.00
GLY	MET	SER	3	4	75.00
GLY	ASN	ALA	6	9	66.67
LYS	ARG	THR	9	14	64.29
GLN	SER	TYR	3	4	75.00
ARG	GLY	ASN	5	7	71.43
SER	GLN	ARG	5	6	83.33
SER	THR	ILE	5	7	71.43
VAL	HIS	ASP	3	4	75.00
VAL	LYS	CYS	5	6	83.33

N_b_: number of occurrence at binding sites; N_t_: total number of occurrence

In Table 2, we have also included the preferred tripeptides at the binding sites of protein-RNA and protein-DNA complexes. The information on tripeptides could identify the binding sites with an accuracy of 78.7% and 71.9% in protein-RNA and protein-DNA complexes. We have compared the preferences of tripeptides at the interface of protein-protein, protein-RNA and protein-DNA complexes. Interestingly, the preferred tripeptides are unique to protein-protein complexes and none of the tripeptides are common with any of the other complexes. This result reveals the existence of different mode of recognition for the protein complexes with other biological molecules.

### Importance of sequence specificity revealed from dipeptide preferences

We have analyzed the preference of residues paired with binding site residues on both N and C directions. The preferred residue pairs with *B binding motifs are DW, CW, MW, CM, CY, MR, MY, PF, PH, QW, SW, TH and WN. On the other hand the preferred residue pairs with B* motifs are CW, HH, IW, MW, QM, RR, TF, WG, WH, WM, WN, YA and YG. Further analysis on the preference of residues on N and C directions of binding sites revealed that the paired amino acids are different on both sides. Specifically, the residues at the N- direction of binding site Trp residue are Ala, and Asp whereas at the C-side are Met, His, Phe, Asp and Val. This result indicates the importance of sequence specificity for binding in protein-protein complexes.

We have compared the specific preferences of dipeptides in protein-protein, protein-RNA and protein-DNA complexes and the topmost five residue pairs are listed in Table 3. In this table, we included the data obtained with the motifs *B and B*. We observed that the residues mainly paired with Trp at the binding sites in protein-protein complexes. On the other hand, the residues preferred to have pairs with Arg and His in protein-RNA complexes. Interestingly, eight out of ten pairs prefer the residue Arg at the binding sites in protein-DNA complexes. This shows the different features of residue pairs at the binding sites for the proteins complexed with proteins, RNA and DNA. Further, we noticed that the residue pair Cys-Trp is common to all the three complexes, which may be a unique pair for binding.

**Table 3:** Topmost five preferred residue pairs at the binding sites of protein-protein, protein-RNA and protein-DNA complexes

Protein-protein		Protein-RNA		Protein-DNA	
*B					
ASP	TRP	CYS	TRP	SER	ARG
CYS	TRP	HIS	ARG	GLY	ARG
ILE	TRP	ASN	ARG	LYS	ARG
MET	TRP	ILE	TYR	ARG	ARG
MET	TYR	TRP	ARG	CYS	TRP
B*					
CYS	TRP	HIS	TRP	ARG	SER
MET	TRP	HIS	HIS	ARG	GLY
TRP	PHE	HIS	ARG	ASN	TRP
TRP	HIS	LYS	HIS	ARG	LYS
TRP	MET	MET	TRP	ARG	ASN

B: Binding. The binding residue are underlined.

### Conformational switching upon complex formation

**Figure 2:**
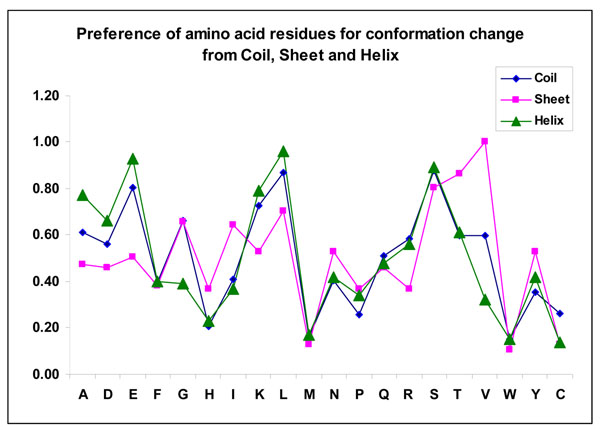
Preference of amino acid residues for conformational change at different secondary structures.

We have analyzed the residues that change their conformation upon binding. We noticed that approximately 7% of residues are involved in conformational changes. The analysis on different secondary structures showed that the changes between regular structures are not favorable, for example, helix to strand and vice versa. Most of the changes are associated with irregular shape or coil. We have also analyzed the preference of amino acid residues to change their conformations upon binding in three different secondary structures. The results are shown in Figure 2.

We noticed that the residues Lys, Glu, Leu and Ser have high preference to change their conformation from helix to coil/strand as well as from coil to helix/strand. Further, Leu, Ser, Thr and Val prefer to change their conformation from strand to coil/helix. The analysis of binding site residues showed that the percentage of residues that change their conformation from coil to strand, coil to helix, strand to coil and helix to coil are, 39%, 17%, 17% and 26%, respectively. This result indicates that the proteins tend to form regular secondary structures upon binding, which agrees with the analysis that the binding site residues are located mainly in helix/strand regions compared with coil [22]. Further, the conformation changes may be necessary for the recognition of protein-protein complexes [23].

We have also analyzed the preference of conformational changes for the neighboring residues of binding sites, N-2, N-1, C+1 and C+2 positions. Interestingly, the preference of next residues (C+1 and N-1 positions) are higher than other positions and the change at the second position on both directions (N-2 and C+2) are similar.

## Conclusions

We have developed an energy based approach for identifying the binding sites in protein-protein complexes. The binding sites identified have been further analyzed based on different sequence and structure based parameters, conservation score, conformational switching and preference of neighboring residues. We observed that the binding residues are significantly highly conserved than the non-binding residues. We have also explored the preferences of residues at the vicinity of binding sites, which showed the importance of sequence specificity. Further, we have analyzed the importance of conformational changes upon complex formation. We noticed that the residues Ser, Leu, Lys, Glu, Thr and Val prefer to change their conformation upon binding. The information obtained in the present study will be useful for understanding and predicting the binding sites of protein-protein complexes.

## Authors' contributions

MMG and KF designed the project. MMG carried out the computations on sequence and structural features. SS and NS are involved in conformational switching. BJ contributed in discussions.

## Competing interests

The authors declare that they have no competing interests.
